# Insurance Issues as Secondary Stressors Following Flooding in Rural Australia—A Mixed Methods Study

**DOI:** 10.3390/ijerph19116383

**Published:** 2022-05-24

**Authors:** John W. McKenzie, Jo M. Longman, Ross Bailie, Maddy Braddon, Geoffrey G. Morgan, Edward Jegasothy, James Bennett-Levy

**Affiliations:** 1 University Centre for Rural Health, The University of Sydney, 61 Uralba Street, Lismore, NSW 2480, Australia; jo.longman@sydney.edu.au (J.M.L.); ross.bailie@sydney.edu.au (R.B.); maddy.braddon@sydney.edu.au (M.B.); geoffrey.morgan@sydney.edu.au (G.G.M.); edward.jegasothy@sydney.edu.au (E.J.); james.bl@sydney.edu.au (J.B.-L.); 2 Office of the Vice Chancellor, Southern Cross University, Lismore, NSW 2480, Australia; 3 Sydney School of Public Health, The University of Sydney, Lismore, NSW 2480, Australia

**Keywords:** disaster recovery, secondary stressors, insurance dispute, mental health, flood, mixed methods

## Abstract

Flood events can be dramatic and traumatic. People exposed to floods are liable to suffer from a variety of adverse mental health outcomes. The adverse effects of stressors during the recovery process (secondary stressors) can sometimes be just as severe as the initial trauma. Six months after extensive flooding in rural Australia, a survey of 2530 locals was conducted focusing on their flood experiences and mental health status. This mixed methods study analysed (a) quantitative data from 521 respondents (21% of total survey respondents) who had insurance coverage and whose household was inundated, 96 (18%) of whom reported an insurance dispute or denial; and (b) qualitative data on insurance-related topics in the survey’s open comments sections. The mental health outcomes were all significantly associated with the degree of flood inundation. The association was strong for probable PTSD and ongoing distress (Adjusted Odds Ratios (AORs) with 95% confidence intervals 2.67 (1.8–4.0) and 2.30 (1.6–3.3), respectively). The associations were less strong but still significant for anxiety and depression (AORs 1.79 (1.2–2.7) and 1.84 (1.2–2.9)). The secondary stressor of insurance dispute had stronger associations with ongoing distress and depression than the initial flood exposure (AORs 2.43 (1.5–3.9) and 2.34 (1.4–3.9), respectively). Insurance was frequently mentioned in the open comment sections of the survey. Most comments (78% of comments from all survey respondents) were negative, with common adverse trends including dispute/denial, large premium increases after a claim, inconsistencies in companies’ responses and delayed assessments preventing timely remediation.

## 1. Introduction

One of the many consequences of global warming is a dramatic increase in the frequency and severity of extreme weather events, including flooding [1]. Being flooded at home can be a traumatic experience; physical injury, destruction of property, dislocation and severe disruption are all common consequences. Understanding the impact of flooding events and implications for the recovery process is increasingly important.

The effects of flooding on mental health are no less important than the immediately obvious physical consequences. For example, in the UK, adverse mental health outcomes have been shown to account for 80% of all Disability Adjusted Life Years attributable to floods [2]. Adverse mental health outcomes associated with floods include PTSD, anxiety, distress and depression [3,4].

### 1.1. Secondary Stressors and Mental Health

After disasters such as flooding, restoration of the built environment, finances and social cohesion can take considerable time and effort. During this time, flood-affected people are especially vulnerable to “secondary stressors” [5,6].

We refer to the immediate and direct adverse effects of disasters such as flooding as primary stressors. The definition of secondary stressors in the research literature has not always been clear or consistent [7]. For the purposes of this paper, we consider secondary stressors to be indirect consequences of a traumatic event (such as flooding) that are ongoing, unresolved obstacles to a return to what is perceived as normality [4,7,8]. Secondary stressors can occur in the immediate aftermath of the event and persist for months or years afterwards [9,10,11]. Secondary stressors can include delays and disputes when dealing with insurance and construction companies, problems with personal relationships, loss of employment and financial hardship [12]. They are associated with poor mental health outcomes [6,12] and a loss of confidence in services and authorities [3,13]. Secondary stressors can have even greater mental health impacts than the original disaster, according to both quantitative studies [14] as well as self-reported by victims [15].

Since secondary stressors are ongoing, unresolved obstacles to recovery, their mental health effects may be different in nature to those from the dramatic, short-term impact of the original disaster [6,8]. People’s patterns of vulnerability can also change through the disaster and recovery phases [16,17]. This complexity implies that no one measure will provide an adequate depiction of flood-affected people’s mental health. Most disaster studies focus on PTSD as the key mental health outcome measure [9], while depression, distress and anxiety are less frequently studied in the literature.

The current study covers a suite of mental health measures; anxiety, depression, flood-related probable PTSD and ongoing flood-related distress. The mental health effects of flooding severity and insurance dispute (the primary and secondary stressors) are modelled.

Insurance dispute is frequently cited as a particularly significant source of secondary stress [3,7,8,18] and its impact can be severe [11,19]. Common complaints include delays in dealing with claims, the attitude of insurance staff, unreasonable denial or reduction in claims, inconsistent decision making and the stressful claims process [3,8,16,19]. In Australia, many insurers use preferred repairers and suppliers and organize and pay for that directly. Other insurers make a payout to the insured person to fund repairs and goods lost or damaged. In the case of extreme-weather-related events, insurance companies may appoint a loss assessor which can add to delays, particularly when many properties are damaged and there are many claims being processed simultaneously [20].

### 1.2. Flooding in the Northern Rivers Region

The Northern Rivers region of northern NSW, Australia, has a resident population of around 240,000 [21]. The main economic drivers include tourism, retail, human services, horticulture and agriculture [22]. The region includes many areas of relative socioeconomic disadvantage [22,23] and has higher proportions of older people, low-income individuals and people with limited education compared to state and national averages [22,23].

The region is a known hotspot for weather-related extreme events, particularly flooding [23,24]. In early 2017, extreme rainfall from ex-Tropical Cyclone Debbie flooded many regions of the Northern Rivers including the major population centres of Lismore and Murwillumbah. There was extensive damage to housing and infrastructure and major disruption of the affected population.

The current work analyses survey responses collected six months after the devastating flood of 2017. We used a mixed methods approach to investigate insurance issues, including disputes, as secondary stressors.

We analyse the extent to which the primary and secondary stressors impact a variety of mental health measures.

We also report a qualitative analysis of how participants’ experiences with insurance acted as a secondary stressor and how this influenced community perceptions of insurance.

## 2. Methods

### 2.1. The Community Recovery after Flood Survey

Six months after the 2017 floods, the University Centre for Rural Health (UCRH, the University of Sydney, Australia) undertook a cross-sectional survey exploring participants’ experience of the flood and their mental health outcomes. The research approach was based on strong community–academic partnerships, purposively surveying a broad cross-section of the community, with recruitment strategies which leveraged these community partnerships supplemented by local media advertising, posters and flyers and an extensive social media strategy. Recruitment focused on hard-to-reach population groups and groups known to be particularly at risk of the effects of extreme-weather-related events, including young people (16–25), older people (75 years and over), those socioeconomically disadvantaged, those identifying as Aboriginal and/or Torres Strait Islander, farmers and business owners. The study did not, therefore, recruit a random and representative sample, as its aim was to describe the associations of exposure to the flood and mental health outcomes rather than to assess prevalence of either flood exposure or outcomes [25].

The survey was made available online, mobile phone and in paper form from September to November 2017 to people aged 16 and over, living in the Northern Rivers at the time of the flood. The questionnaire included a variety of exposure measures, such as inundation of home, evacuation and impact on respondents’ neighbourhood and family.

Three specific questions about insurance were included (see Appendix A for the relevant survey questions):

- Whether respondents felt that they got the support they needed from their insurance company (with an N/A response taken to mean the respondent had no insurance or no need to claim);
- Whether they believed that they were fully insured but the insurance company rejected or disputed their claim;
- Whether insurance companies were to blame for anyone’s distress after the flood.

There were also eight opportunities for open comment (free text).

### 2.2. Quantitative Methods

A total of 2530 surveys were returned. 

The quantitative study cohort consisted of all survey respondents who:

- Reported being flooded in their household (1215 retained);
- Gave an answer other than N/A in the insurance support question, indicating that they had dealings with an insurance company after the flood (697 retained);
- Did not select “Other” for housing type (response options were Rent, Mortgage, Own and Other) (629 retained);
- Had complete data for the demographic variables considered (572 retained) and exposure measures (549 retained);
- Answered all questions used in the analysis for the PTSD and “still distressed” mental health outcomes (521 retained).

Respondents who selected “Other” for housing type were excluded from further analyses because the free text showed they were too diverse to be meaningfully analysed: responses included “staying with family”, “in a retirement home” and “homeless”.

We used anxiety (GAD-2) and depression (PHQ-2), as well as two flood-specific mental health measures “probable PTSD” (PCL-6) and “still distressed” as dichotomous mental health and wellbeing outcome measures. Previously published papers have further details on these measures [25] and their prevalence amongst the survey respondents [26].

Our key secondary stressor exposure was “insurance rejected or disputed” (dispute).

We used self-reported “flooded inside the living area of their house” as a dichotomous degree of inundation variable. (Everyone in the study cohort reported a degree of inundation: in the living and/or non-living areas of their household.).

Consistent with the English National Study of Flooding and Health [18], we developed logistic regression models for the mental health outcomes. We regarded the primary and secondary stressors (degree of inundation and insurance dispute) as the primary predictors and adjusted for demographics (employment status, relationship status and housing type). These were selected from a broader suite of demographic factors (including age and gender) after a process of stepwise regression.

### 2.3. Qualitative Methods

The free text responses from all 2530 completed surveys were analysed. Using a qualitative descriptive approach [27], qualitative data from the eight free text opportunities in the survey were deductively coded using a realist content analysis following the steps outlined by Elo and Kynga [28]. An initial structured coding index was generated based on the wider literature on mental health and floods [3,5,18], the interests of the community which were established through our community–academic partnership and the aims of this paper. Two authors (J.L. and M.B.) trialed the index by independently coding a sample of comments from participants that were particularly extensive and detailed, and the coding index was refined following discussion and agreement between J.L. and M.B. This process was repeated with a further sample of comments and coding (independently by J.L. and M.B.) was tested for concordance in NVivo 11, showing a high level of concordance. J.L., M.B. and J.M. then used the final index to code all the data. Once the data were coded, the codes were grouped together into categories and the interpretation developed by J.L. and M.B. as described below along with the selection of illustrative quotes from the raw data.

## 3. Results

### 3.1. The Quantitative Study Cohort

A total of 2530 surveys were returned. A separate sub-study in which we door-knocked in flood-affected and non-flood-affected areas showed that around 5% of the population in both the flooded areas and those outside had completed the survey [25].

Of 2530 questionnaires returned, 1215 respondents reported that they were flooded in their household. Of these, 518 were excluded from further analysis because they responded that insurance support was not applicable, leaving 697 candidate surveys. The final quantitative cohort was comprised of the 521 surveys that had sufficiently complete demographic, primary mental health outcomes (PTSD and ongoing distress) and exposure data.

Table 1 below provides summary information about this cohort. They broadly resemble the total sample of 2530 respondents although certain groups of respondents are somewhat under-represented in the final study cohort: for example, being in a relationship (63.5% in the study cohort compared to 67.7% of all surveys) and employed (63.5% compared to 68.6%). This is consistent with the finding that respondents with various risk factors, including low socioeconomic status, were especially impacted by the 2017 floods [23]. House insurance is compulsory for mortgagees, who are therefore also over-represented, comprising 37.0% of inundated households and 44.9% of the study cohort. The demographic profiles of the survey respondents are presented in more detail in a previous paper [25].

### 3.2. Quantitative Results

Table 2 below lists the prevalence of various exposures and mental health outcomes amongst the 521 quantitative cohort members. A total of 96 respondents from the study cohort reported insurance denial, 159 had probable PTSD and 224 reported ongoing distress from the floods. We used “flooded inside house” in our regression models as the measure of severity of flooding.

The logistic regression results adjusted for the retained demographic factors are in Table 3.

The degree of inundation was significantly associated with each mental health outcome, strongly so for PTSD (AOR 2.67 (1.8–4.0)) and ongoing distress (AOR 2.30 (1.6–3.3)).

The association between insurance dispute and ongoing distress was very strong (AOR 2.33 (1.5–3.7), as was the association between insurance dispute and probable depression (AOR 2.49 (1.5–4.1)). Dispute was also significantly associated with anxiety (AOR 1.82 (1.1–3.0)) but not with PTSD (AOR 1.40 (0.86–2.3)). It is striking that the associations of dispute with ongoing distress and depression were as strong or even stronger than their associations with the flood exposure measure.

As expected, some demographics appear to be risk factors. Being unemployed was strongly associated with higher rates of anxiety (AOR 2.12 (1.4–3.3)) and depression (AOR 1.82 (1.2–2.9)), while not being in a relationship was associated with higher rates of depression (AOR 1.91 (1.2–3.0)). Gender and age factors were not significant and were eliminated during the stepwise regression process.

### 3.3. Qualitative Results

This section analyses free text comments about insurance from all 2530 surveys that were returned.

#### 3.3.1. Context for the Qualitative Results

Amongst the 2468 (95.8%) survey respondents who answered the question, 166 (6.7%) “believed that they were fully insured but the insurance company rejected or disputed their claim”. Of respondents who answered, 49% believed insurance companies were “a lot” or “entirely” to blame for anyone’s distress after the flood, while 49% of those with insurance reported that they did not get the support they needed from their insurance company.

Of eight free text opportunities, 2114 respondents (84%) wrote in at least one. Insurance was raised by 14.8% of respondents (381 comments from 312 respondents). This compares with, for example, the topic of disaster relief measures which received 198 comments. A majority (78%) of the comments about insurance were negative. This was the case even when the respondent had not had an insurance dispute themselves. Respondents also described how problems with insurance companies linked to other secondary stressors such as financial stress, loss of local businesses, breakdowns in relationships and decreases in the value of “home”.

The 521 respondents in the quantitative cohort made similar comments but were more negative: of 251 who entered free text, 61 included insurance comments and all but 3 were negative.

Respondents described several issues with insurance companies and how these impacted their mental health.

#### 3.3.2. Access to Insurance and Clarity of What Was Covered

Respondents commonly reported difficulties with access including availability, affordability and a lack of clarity in insurance companies’ communications leading to uncertainty about what was covered by their insurance. At the heart of these comments was a reported lack of clarity around how the “event” was labelled and associated disparities within and between insurance companies about if and how they provided support following the flood, i.e., some insurers labelled the event as a “flood” and would not therefore support claims for water damage unless respondents had “floods” included as part of their insurance policy, whereas others labelled it as “storm” and therefore supported claims for damage even when flood insurance was not part of the insurance policy.

“Govt declared the damage to be storm-related, not a flood, yet my [insurance company] refused to help. I was not insured for flood cover as it was too expensive.”

(No. 1100, disputed)

Respondents described how this linked to negative mental health effects, for example:

“It has been very demoralising watching all other homes in the street being repaired by their insurance companies for storm damage, whilst we live in our flood damaged home, with no floor covering, cut out walls, kitchen, laundry and bathroom all in need of repairs”

(No. 909, disputed)

#### 3.3.3. Claims Handling and Dispute Resolution—Customer Care

Some respondents described the poor attitude of insurance company staff, which had the potential to negatively impact on mental health. For example:

“Insurance companies appear to have the attitude that you as a claimant are somehow to blame for the problem … If you don’t know the right question then you never get a satisfactory answer. And once again it’s your fault. I had a very nasty experience when a staff person from the assessor company told me to go and buy a lottery ticket.”

(No. 1932, not disputed)

Respondents who had not necessarily experienced an insurance dispute themselves described the poor behaviour of some insurance companies using emotive language, suggesting distress experienced vicariously in the community rather than grounded in personal experience. For example:

“…the impact from the lack of empathy and response from companies such as [insurance company] is disgusting, the way they have treated their customers and elderly who have no way of defending themselves is despicable.”

(No. 1176, not disputed)

Respondents who had had to deal with insurance companies reported some difficulties with effective communication, including: companies being unresponsive and/or unreliable in their communications; having to tell their “story”, being asked for new requirements or being told different answers each time they contacted the insurance company; and how stressful they found it liaising with insurance companies and/or practical issues with communicating (such as a hearing impairment), in particular linked to vulnerabilities such as pre-existing anxiety or living with a physical disability. Such difficulties were linked by respondents to exacerbations of existing or new mental health issues.

“My husband has dementia he’s 71. My mother is 89 and has mobility problems. I’m hearing impaired. Dealing with insurance claims … was extremely difficult … I was frustrated by people who don’t return calls/messages, say they would come and didn’t or often didn’t feel validated (others worse off etc). I now suffer anxiety.”

(No. 1677, not disputed)

Respondents suggested that assistance with communicating with insurance companies would be welcomed both in terms of general communication and in dispute situations, for example:

“It would be fantastic if there was someone able to help you clearly assess monetary values of losses and/or be there beside you in conversations with the insurance companies as you negotiate your way through the process.”

(No. 94, not disputed)

and

“I wasn’t covered for flood, however in my opinion it was initially caused by a storm, my claim was rejected and I didn’t have the energy, as I get very anxious dealing with things like this, and it all became too daunting to deal with on my own. I would have liked someone to really help me fight the claim!!”

(No. 1377, disputed)

#### 3.3.4. Claims Handling and Dispute Resolution—Delays

Respondents commonly reported significant delays in claims handling. These delays occurred at multiple points.

Firstly, there were delays prior to assessment of a claim when respondents were waiting for assessors to inspect their property (photographic evidence was often deemed insufficient) and expected to leave the damage and live/work around it somehow, for example for “… several weeks after the event” (No. 1698, not disputed). Respondents expressed distress about the additional damage, both physical and emotional, caused due to these delays. Some respondents, for example, found themselves living in insanitary conditions with mould. In terms of their mental health, delays meant living in a state of “limbo”, unable to get on with their lives or recover.

Secondly, respondents reported delays before a decision was made by insurance companies about a payout.

“… applications were lost, delaying any help for many weeks. This was a greater mental stress than the physical clean up”

(No. 1880, disputed)

A third point of delay was once a decision was made, in actually paying out, and finally, in organising repairs.

“The Insurance company took 6 months to repair and caused more pain and stress”

(No. 1479, not disputed)

#### 3.3.5. Links between Insurance Problems and Other Secondary Stressors

Respondents linked problems with insurance companies to other secondary stressors such as financial stress, loss of local businesses, breakdowns in relationships and decreases in the value of “home” (both fiscally and emotionally/psychologically) and community.

“The inequity in the insurance companies’ treatment of victims has led to a lot of unrest in the community and bad feelings still exist between neighbours. The social effects on the population of our small village will be felt for a long time to come.”

(No. 175, not disputed)

## 4. Discussion

The quantitative and qualitative data from the present study clearly demonstrate that adverse experiences with insurance companies at a time of particular and intense vulnerability can act as a substantial secondary stressor.

We found especially strong associations between the secondary stressor (insurance dispute) and both depression and ongoing distress, while the primary stressor (degree of inundation) was more strongly associated with probable PTSD.

Other studies have reported the negative mental health impacts of insurance disputes after flooding events. In particular, the UK National cohort study [4,10,18] investigated the effects of flooding on a variety of mental health outcomes. Mulchandarni et al. [18] specifically studied the effect of insurance as a secondary stressor using data from a survey 2 years after flooding. Of their three outcome measures (depression, anxiety and PTSD), only PTSD was significantly associated with insurance dispute (AOR 2.54 (1.1–5.9)), and their associations with severe insurance stress were marginally significant (AORs 11.08 (1.11–110.3), 4.48 (1.0–19.7) and 7.95 (2.1–30.1), respectively; see [18] Table 3).

The qualitative data support the quantitative data in linking insurance disputes with negative mental health outcomes and impeded recovery. Reported difficulties included affordability, failure to compensate, lack of clarity and consistency in insurance policies and customer care including claim handling, dispute resolution and delays. Some respondents noted explicit links with other secondary stressors including financial stress and relationship breakdowns. Adverse opinions of the insurance industry were frequently reported by respondents on the basis of others’ experiences; these opinions were only weakly reflected in the quantitative data on insurance dispute.

These findings have implications for clinical practice, recovery support and conceptual understanding. The highly significant association between insurance dispute and ongoing distress suggests that respondents still engaged with insurance companies at 6-month follow-ups may be vulnerable to the onset or exacerbation of depression or anxiety disorders if their disputes are not satisfactorily resolved [29]. The distinction between natural distress and adverse mental health outcomes and the difficulty distinguishing them in the aftermath of a natural disaster [9,12] underlines the importance of appropriate follow-up over time so that clinicians can distinguish between those suffering transient distress from those in need of ongoing mental health support.

Our qualitative results suggest possible improvements to the support available during recovery. As in previous studies [5,16], we find that the stress of managing one’s own recovery and dealing with applications for support, insurance and other bureaucracies may itself be a significant secondary stressor. Appropriate administrative support from experienced personal “advocates” for the completion of these tasks may mitigate the stress they cause and improve their completion and success rates. There are support services provided by the Insurance Council of Australia and Legal Aid, but clearly many consumers were unaware or unable to access them.

It may also be necessary to introduce “standard” insurance policies similar to the practice of standardised tenancy leases, that cover all damage sustained due to government-declared disasters.

Delayed recovery as a result of ongoing insurance dispute is a strong theme in our qualitative data and in previous studies [7,9,11]. The process may be expedited by a policy that allows clean-up and repairs to commence once the damage has been properly documented.

Conceptually, our results suggest distinct trauma mechanisms for flood-affected individuals who are in dispute with insurance companies: the primary (flood event) and secondary (in our case insurance dispute). In the first instance, there may be a primary traumatic impact in the early months from a high level of flood exposure, resulting in PTSD and anxiety. The trauma may cause PTSD symptoms such as flashbacks and nightmares, and anxiety about future occurrences, housing and work issues and financial problems. Later, there may be a secondary impact of insurance disputes and rejected claims, which if not rapidly resolved may manifest primarily as ongoing distress, depression and anxiety.

Depression is often associated with experiences of loss or deprivation [30]. It is therefore a likely consequence of insurance disputes and rejection which involve enduring property damage and financial or property loss. Anxiety is typically associated with future threat [30]. It is therefore a likely consequence of the threat of ongoing financial and housing issues. The qualitative responses support these interpretations in identifying financial losses, delays in a return to normalcy and uncertainty of outcome as key influences on the mental health and wellbeing of respondents.

Our results also point to a lack of effective policy responses to ongoing disaster insurance issues. In 2011, the Australian Government undertook a review of disaster insurance [31]. The findings from the present study highlight that many of the issues identified during the 2011 review remain problematic many years later, including affordability, clarity of coverage, consumer awareness, claims handling, dispute resolution and delays.

Insurance companies should also note that, after a disaster, a relatively small number of rejected or disputed claims can translate into broad community disaffection with the services they provide.

### Strengths and Limitations

This work contributes in several ways to our understanding of recovery from flooding, and the potential for insurance disputes to significantly impede that recovery. The reporting of four distinct mental health outcomes provides insights into the complexity of individuals’ responses to both the primary and secondary stressors we studied. Furthermore, the current work appears to be the only study which has collected such survey data in the first year post-flood or combined quantitative and qualitative data to articulate the particular concerns of affected individuals. There are some limitations that should be noted. The quantitative study cohort could be better defined. We had no direct survey response that participants made a flood-related insurance claim, only that they were inundated and did not deny having coverage.

One outcome measure is overly broad. The “ongoing distress” survey item is likely to capture a broad range of circumstances from adverse mental health outcomes to frustration and anger as recovery is delayed or denied. It would have been useful to include more items to further understand its contributing factors.

The small numbers preclude a thorough analysis of possible confounding from associations between demographic factors and insurance dispute. In particular, homeowners and older respondents had the lowest dispute rate. STATA’s margins command revealed no substantial confounding from these sources.

## 5. Conclusions

This work informs our understanding of recovery from flooding in several ways. The distinct patterns of influence of the primary and secondary stressors on the four mental health outcomes have sketched the complexity of these stressors’ impacts on mental health and provided some tentative first steps towards a causal model.

There are implications for counsellors and support workers, who should be mindful of the unfolding of mental health responses to flooding trauma and subsequent secondary stressors such as insurance dispute. In the medium term, a lack of return to normalcy is likely to be a sign that possible distress and depression need to be monitored, including amongst those who appeared to cope well with the initial disaster.

Finally, this work underlines the need for clarity in insurance coverage as well as for support in the management of victims’ disaster recovery.

It remains a profound irony that processes designed to support recovery, such as home and contents insurance, can in practice act as serious additional stressors.

## Figures and Tables

**Table 1:** Demographics of the quantitative study cohort (*n* = 521) and all surveys that completed the item.

	Quant Cohort	All Surveys	
Variable	Value	Number (%)	
Age	Under 30	32 (6.1)	197 (8.4)
	31–64	401 (77.9)	1711 (72.8)
	Over 64	88 (16.9)	442 (18.8)
Gender	Female	363 (69.7)	1617 (68.4)
Employed	Yes	335 (63.5)	1612 (68.6)
In Relationship	Yes	331 (63.5)	1581 (67.7)
Housing Status	Renting	117 (22.5)	596 (26.5)
	Mortgage	234 (44.9)	888 (39.4)
	Owner	170 (32.6)	767 (34.1)

**Table 2:** Prevalence of exposures and mental health and wellbeing outcomes.

Variable	Number (%)
Exposures:	
Insurance Dispute	96 (18.4)
Flooded Inside	279 (53.6)
Mental Health:	
Probable PTSD	159 (30.5)
Anxiety ^1^	142 (27.7)
Depression ^1^	134 (26.1)
Still Distressed	224 (43.0)

^1^ The anxiety and depression measures have some missing values (9 and 8, respectively).

**Table 3:** Adjusted regression results.

	Condition			
	PTSD	Anxiety	Depression	Still Distressed
**Co-Variate**	**Adjusted Odds Ratios (95% CI)**			
Insurance Dispute	1.40 (0.86–2.3)	1.82 * (1.1–3.0)	2.49 ** (1.5–4.1)	2.33 *** (1.5–3.7)
Flooded in House	2.67 *** (1.8–4.0)	1.79 ** (1.2–2.7)	1.80 ** (1.2–2.8)	2.30 *** (1.6–3.3)
Unemployed	1.73 * (1.1–2.7)	2.12 *** (1.4–3.3)	1.82 ** (1.2–2.9)	1.47 (0.98–2.2)
Not in a relationship	1.45 (0.96–2.2)	1.04 (0.67–1.6)	1.91 ** (1.2–3.0)	0.35 (0.82–2.2)
Housing Status:				
Renting ^1^	1.76 * (1.0–3.0)	2.27 ** (1.3–3.9)	1.45 (0.83–2.5)	1.35 (0.82–2.2)
Mortgage ^1^	1.33 (0.82–2.2)	1.28 (0.77–2.1)	0.868 (0.52–1.5)	1.36 (0.87–2.1)
Adjusted R^2^	0.076	0.070	0.106	0.071

Significance levels: * 5%, ** 1%, *** 0.1%. ^1^ Compared to homeowners.

## Data Availability

The datasets used and analysed during the current study are available from The University Centre for Rural Health on reasonable request.

## Appendix A. Relevant Survey Items

*Free Text:*

1. Is there anything on your mind that you want to say right upfront about the heavy rain and flood in March/April 2017?
6. Did you have to evacuate your home?
  Is there anything more you want to say about this?
9. Did you have to evacuate the business you own or the place where you work?
  Is there anything more you want to say about this?
15. In your view, are any of the following organisations [Insurance company] to blame for anyone’s distress after the flood?
  Is there anything more you want to say about this?
16. Were you in the Northern Rivers when the heavy rain fell in June 2017 (about 3 months after the March/April flood)?
  If Yes, did this affect you in any way? If so, how?
45. Thinking back, have the severe rain and flood resulted in you being able to make any positive changes in your life?
  If yes: Could you give an example of your positive changes?
58. Is there anything else you want to add about your experience of the March/April flood or what things are like for you now?
  Feedback: If you have any queries, suggestions or feedback, please use the space below (you can use the space on the back page if you need more room).

*Event Specific Multiple Choice:*

4. Were non-liveable areas of your home damaged or flooded (e.g., garage, garden shed)?
  Options: Yes/No
5. Was at least one liveable room in your home damaged or flooded (e.g., bedroom, living room, kitchen, bathroom)?
  Options: Yes/No
11. Did any of the following happen at the time of the March/April flood or afterwards? (Please tick all that apply)
  You believed you were fully insured but the insurance company rejected or disputed your claim
14. After the March/April flood, did the support you requested or received from the following organisations or groups meet your needs?—Insurance company
  Options: No/Partially met my needs/Fully met my needs/Don’t know/N/A
15. In your view, are any of the following organisations to blame for anyone’s distress after the flood?—Insurance company
  Options: Not at all/Partly/A lot/Entirely

*Demographics:*

26. How old are you? [years]
27. Where were you born?
  Options: Born in Australia/Born overseas
28. What language do you speak at home?
  Options: Mainly English/Mainly a language other than English
29. Are you of Aboriginal or Torres Strait Islander origin?
  Options: Yes, Aboriginal/Yes, Torres Strait Islander/Yes, both Aboriginal and Torres Strait Islander/No
30. Are you…?
  Options: Female/Male/Other
31. Do you consider yourself to be:
  Options: Lesbian, gay or homosexual/Straight or heterosexual/Bisexual/Queer/Transgender/Other
32. What is your relationship status?
  Options: Single/Married or other formal commitment/In a relationship but not living together/Living together (in a defacto relationship)
34. Have you completed any formal education? (Please tick all that apply)
  Options: Year 10 certificate (or equivalent)/Year 12 certificate (or equivalent)/Diploma or trade (e.g., child care, hairdresser, chef)/University degree/None of the above/Other (please specify)
35. Are you currently in paid work? (Please tick all that apply):
  Options: Part-time work/Full-time work/No/Casual work (hours vary & are not set)/Shift-work
37. What was your housing situation at the time of the March/April 2017 flood?
  Options: Renting/Had a mortgage/Owned home outright/Other (please specify):
40. At the time of the March/April flood, were you receiving any income support from the government? (Please tick all that apply)
  Options: Age-related pension/Youth allowance/Newstart/Disability support pension/Parenting payment/None of these/Other (please specify):
42. What is your approximate total household income per year before tax?
  Options: Under $50,000/$50,000–$100,000/Over $100,000/Prefer not to answer
